# Photodynamic Inactivation of Herpes Simplex Viruses

**DOI:** 10.3390/v10100532

**Published:** 2018-09-29

**Authors:** Andrea L.-A. Monjo, Eric S. Pringle, Mackenzie Thornbury, Brett A. Duguay, Susan M. A. Monro, Marc Hetu, Danika Knight, Colin G. Cameron, Sherri A. McFarland, Craig McCormick

**Affiliations:** 1 Department of Microbiology and Immunology, Dalhousie University, 5850 College Street, Halifax, NS B3H 4R2, Canada; andrea.monjo@dal.ca (A.L.-A.M.); eric.pringle@dal.ca (E.S.P.); mackenzie.thornbury@umontreal.ca (M.T.); bduguay@dal.ca (B.A.D.); danika.knight@dal.ca (D.K.); 2 Department of Pathology and Cell Biology, University of Montreal, V-541 Pavillon Roger Gaudry, 2900 Boulevard Édouard-Montpetit, Montreal, QC H3C 3J7, Canada; 3 Department of Chemistry, Acadia University, 6 University Avenue, Wolfville, NS B4P 2R6, Canada; 053189m@acadiau.ca (S.M.A.M.); marc@photo-dynamic.com (M.H.); 4 Photodynamic, Inc., 1344 Summer Street, Halifax, NS B3H 0A8, Canada; cgcamero@uncg.edu; 5 Department of Chemistry and Biochemistry, University of North Carolina at Greensboro, 301 McIver Street, Greensboro, NC 27402, USA

**Keywords:** HSV-1, HSV-2, photodynamic inactivation, plaque assay, natural product, antiviral

## Abstract

Herpes simplex virus (HSV) infections can be treated with direct acting antivirals like acyclovir and foscarnet, but long-term use can lead to drug resistance, which motivates research into broadly-acting antivirals that can provide a greater genetic barrier to resistance. Photodynamic inactivation (PDI) employs a photosensitizer, light, and oxygen to create a local burst of reactive oxygen species that inactivate microorganisms. The botanical plant extract Orthoquin^TM^ is a powerful photosensitizer with antimicrobial properties. Here we report that Orthoquin also has antiviral properties. Photoactivated Orthoquin inhibited herpes simplex virus type 1 (HSV-1) and herpes simplex virus type 2 (HSV-2) infection of target cells in a dose-dependent manner across a broad range of sub-cytotoxic concentrations. HSV inactivation required direct contact between Orthoquin and the inoculum, whereas pre-treatment of target cells had no effect. Orthoquin did not cause appreciable damage to viral capsids or premature release of viral genomes, as measured by qPCR for the HSV-1 genome. By contrast, immunoblotting for HSV-1 antigens in purified virion preparations suggested that higher doses of Orthoquin had a physical impact on certain HSV-1 proteins that altered protein mobility or antigen detection. Orthoquin PDI also inhibited the non-enveloped adenovirus (AdV) in a dose-dependent manner, whereas Orthoquin-mediated inhibition of the enveloped vesicular stomatitis virus (VSV) was light-independent. Together, these findings suggest that the broad antiviral effects of Orthoquin-mediated PDI may stem from damage to viral attachment proteins.

## 1. Introduction

Herpes simplex viruses are ubiquitous and cause life-long infections of their human hosts. HSV infections are primarily acquired at mucosal surfaces where initial epithelial cell infections can lead to disease manifestations that include herpes labialis, genital herpes, gingivostomatitis, herpetic whitlow, encephalitis, aseptic meningitis, neonatal herpes, and stromal keratitis [1]. HSV-1 and HSV-2 share common structural features, including a large double-stranded DNA genome protected by a nucleocapsid, a tegument layer containing viral proteins that are delivered to the host cell early in infection, and an envelope derived from host membranes studded with viral glycoproteins that facilitate attachment and entry into host cells. While HSV-1 and HSV-2 are similar, they share only ~55% nucleotide identity. 

Lytic HSV infection of a cell begins with initial attachment to negatively charged cell surface heparan sulfate and chondroitin sulfate glycosaminoglycans, followed by stable attachment of viral glycoproteins to cell surface receptors and fusion of the viral envelope to the plasma membrane or internal membranes [2]. Fusion of viral and host membranes enables delivery of the viral nucleocapsid and tegument proteins to the cytoplasm. After the HSV nucleocapsid arrives at the nucleus and delivers the viral genome, HSV genes are expressed in an ordered temporal cascade. Viral egress requires primary envelopment of the genome-containing capsid at the inner nuclear membrane, fusion with the outer nuclear membrane and final envelopment at the trans-Golgi network. Mature virions then exit through the cellular secretory system. 

All stages of the HSV replication cycle offer potential targets for direct-acting antiviral (DAA) therapy. However, DAAs can be undermined by the emergence of drug resistant mutant viruses. Anti-HSV DAAs acyclovir and foscarnet offer examples of the hazards of drug resistance. These drugs interrupt HSV genome replication by different mechanisms of action. Acyclovir is a nucleoside analogue prodrug that selectively targets HSV infected cells through the action of the HSV thymidine kinase (TK) enzyme, which efficiently phosphorylates acyclovir and facilitates incorporation into nascent viral DNA genomes by the HSV DNA polymerase enzyme; incorporation of acyclovir into viral DNA terminates genome replication [3]. By contrast, the pyrophosphate analogue foscarnet blocks viral genome replication by directly inhibiting HSV DNA polymerase activity [4]. Though resistance to acyclovir occurs at a low prevalence (≤1%) in immunocompetent patients [5], immunocompromised patients experience much higher rates (4–10%) [6]. Unsurprisingly, resistance to acyclovir, and its derivatives, and to foscarnet, has been mapped to mutations in the viral thymidine kinase and the DNA polymerase catalytic subunit, respectively [7]. Currently, Abreva (n-docosanol) is the only over-the-counter topical antiviral for herpes labialis. Abreva is incorporated into the cellular plasma membrane and prevents fusion steps essential for HSV-1 entry into epithelial cells [8]. However, it must be applied repeatedly throughout the day due to rapid plasma membrane turnover and the subsequent loss of n-docosanol [9]. This host-targeted antiviral mechanism prevents development of viral resistance, but Abreva is only approved for perioral lesions, so there is a still a gap in treatment for lesions in other areas of the body. 

Like Abreva, there is potential for more broadly-acting antiviral drugs that exploit the physical structure of the HSV virion and block early stages of infection, such as attachment and penetration. In this study, we explored the potential of the plant-derived photosensitizing agent Orthoquin^TM^ to mediate photodynamic inactivation (PDI) [10] of virions. The clinical utility of Orthoquin PDI of oral biofilms is an area of active investigation and there is clear potential for wider range of clinical applications. The basis of PDI is the photodynamic effect, which has been exploited in photodynamic therapy (PDT) [11,12] for treatment of cancers [13] and age-related macular degeneration [14]. In PDT, photosensitizer compounds when exposed to visible light react with oxygen to generate reactive oxygen species (ROS) that include singlet oxygen [15]. ROS damage proteins, nucleic acids and lipids, which can lead to cell death. Some of the most well-known photosensitizing molecules for PDT are based on a tetrapyrrolic core and are found in naturally occurring pigments, such as heme, chlorophyll, and bacteriochlorophyll [16]. The accepted phototoxic mechanism for these types of photosensitizers involves the formation of an excited triplet state upon light absorption that can participate in electron (Type 1) or energy (Type 2) transfer processes. Type 1 electron transfer reactions typically lead to the formation of radical species like superoxide or hydroxyl radicals, whereas Type 2 energy transfer produces cytotoxic singlet oxygen. It is possible for a photosensitizer to initiate both Type 1 and 2 photoprocesses simultaneously, depending on the specific chemistry of the photosensitizing agent and its environment [16]. Due to the high reactivities and short lifetimes of oxidizing molecules, it is expected that only viral structures in close proximity to activated photosensitizing compounds will be affected.

Orthoquin has been shown to have bacteriocidal properties and to disrupt bacterial biofilms without causing overt inflammation [17]. In this study we investigated Orthoquin’s anti-herpesviral properties and mechanism of action. We showed that sub-cytotoxic doses of Orthoquin reduced HSV-1 and HSV-2 plaque formation in a light-dependent manner, whereas high doses displayed light-independent antiviral effects. Surprisingly, HSV-2 displayed high intrinsic photosensitivity, so we focused primarily on the relatively photo-resistant HSV-1. PDI of HSV-1 required close proximity between Orthoquin and the viral inoculum, whereas pre-treatment of target host cells with Orthoquin exposed to light had no effect. High doses of Orthoquin disrupted immunodetection of a subset of HSV-1 structural proteins by a pan-anti-HSV-1 polyclonal antibody, suggesting that PDI may cause physical damage to proteins on the virion exterior that prevents infection. Finally, we demonstrated light-dependent Orthoquin PDI of adenovirus infection and light-independent inhibition of vesicular stomatitis virus (VSV) infection.

## 2. Materials and Methods

### 2.1. Characterization of Orthoquin™

HPLC analysis was performed on an Agilent/Hewlett-Packard 1100 series instrument (ChemStation Rev. A. 10.02 software, Santa Clara, CA, USA) equipped with a UV-Vis detector. Separation was achieved on a Hypersil GOLD C18 (Waltham, MA, USA) reversed-phase column with an A-B gradient (90% → 0% A; A = 0.2% formic acid in H_2_O, B = MeOH). Reported retention times are correct to within ± 0.1 min. Column temperature was recorded to be 35 °C, flow rate was 1 mL min^−1^ using an injection volume of 20 µL for sample prepared at 5 mg mL^−1^. Absorbance values for the samples were recorded at 254 nm, 306 nm, 320 nm, 435 nm, and 450 nm. The constituents giving rise to the largest signals at these wavelengths were identified by analyzing a panel of commercial standards under the same conditions and also by the method of standard addition. The largest signals at these wavelengths were due to polydatin (63 µg mg^−1^), resveratrol (60 µg mg^−1^), anthraglycoside B (12 µg mg^−1^), rhein (5.6 µg mg^−1^), emodin (50 µg mg^−1^), and physcion (10 µg mg^−1^).

### 2.2. Cells, Viruses, and Reagents

HeLa, HEK293A, and Vero (African Green Monkey kidney) cells were cultured in Dulbecco’s modified Eagle’s medium (DMEM, HyClone, Logan, UT, USA) supplemented with 10% fetal bovine serum (FBS, Life Technologies, Carlsbad, CA, USA), 100 U/mL penicillin, 100 µg/mL streptomycin, and 20 mM l-glutamine (Life Technologies). hTert-immortalized human foreskin fibroblast (Tert-BJ, a gift from Dr. William Hahn, Dana-Farber Cancer Institute, Boston, MA, USA) cells were maintained in a 4:1 blend of DMEM and modified Eagle’s medium-199 (Life Technologies) with 15% FBS and 4 mM l-glutamine. Cells were maintained at 37 °C in 5% CO_2_ atmosphere.

We used HSV-1 strain 17syn + and HSV-2 strain 186. Inoculum was generated by infecting a monolayer of Vero cells with a low MOI and harvesting cell-associated virus three days post-infection. Briefly, cells were dissociated from the plastic by pipetting and were separated from the supernatant by centrifugation. The cell pellet was resuspended in serum-free media and lysed with three freeze‒thaw cycles. The lysate was sonicated with a probe sonicator, on ice, and the cellular debris were removed by centrifugation. The supernatant was then aliquoted and frozen at −80 °C. VSV-GFP (a gift from Dr. Roy Duncan, Dalhousie University, Halifax, NS, Canada), was similarly grown in Vero cells and isolated from the supernatant after cells and debris were removed by centrifugation. We used a GFP-Adenovirus 5 vector (AdV-GFP, Clontech, San Diego, CA, USA). This virus lacks the essential E1 early genes, which can be complemented *in trans* by generating the virus in HEK293A cells. Orthoquin was provided by PhotoDynamic Inc. (Lot # MH3-94c, Halifax, NS, Canada).

### 2.3. Cytotoxicity Assay

HeLa and hTert-BJ cells were seeded in 96-well plates at 20,000 and 5000 cells per well, respectively, in duplicate plates. The following day, cells were treated with 100 μL of 2-fold dilutions of Orthoquin (from 2.4 to 78 μg/mL) or with 100 μL of equivalent DMSO concentrations (from 0.78 to 0.024%), in triplicate. After 16 h of Orthoquin treatment, plates were placed 20 cm under a 65 W LED lamp for 30 min at room temperature (“dark” plates were wrapped in aluminum foil) and were returned to the incubator. The total fluence delivered to the light-treated plates was 37.8 J cm^−2^ at a rate of 21 mW cm^−2^. After 48 h of incubation, 10 μL of alamarBlue were added to each well and plates were placed back in the incubator for 3.5 h. Fluorescence was recorded using a Tecan (San Jose, CA, USA) Infinite M200 PRO microplate reader (ex/em: 560/590 nm) and normalized to the equivalent concentration of DMSO. The phototoxic concentration (CC_50_) for each cell line was calculated using Prism 7 (Graph Pad, La Jolla, CA, USA) with a non-linear fit of log (inhibitor) vs. response (variable slope, four parameters).

### 2.4. Photodynamic Inactivation of Viral Inoculum

Unless otherwise stated, Orthoquin and virus preparations or controls were pipetted into clear for “light” (VWR) or black for “dark” (Argos Technologies, Vernon Hills, IL, USA) 1.5 mL polypropylene microcentrifuge tubes, which were placed on their sides under a 65 W visible LED lamp 20 cm from the bulb for 10 min at room temperature (12.6 J cm^−2^, 21 mW cm^−2^). HSV-2 and VSV inocula were exposed to light for only 5 min because they were photosensitive. The temperature of the inoculum under these lighting conditions did not exceed 30 °C. 

### 2.5. Plaque Assays

#### 2.5.1. HSV-1 and HSV-2 Plaque Assays

HeLa cells were seeded at a density of 4.5 × 10^5^ cells/mL in 24-well cluster dishes. The following day, medium was removed and cells were inoculated with 50 μL of PDI-treated or mock-treated HSV-1 or HSV-2 (in serum-free media) for 1 h with shaking every 10 min. We adapted an Avicel, microcrystalline cellulose overlay, first used to plaque influenza [18] to plaque HSV-1 and HSV-2. After infection, the inoculum was rinsed off with 1 mL PBS, and 500 μL of 1.2% Avicel (a gift from FMC Biopolymer, catalogue RC-591, Philadelphia, PA, USA) overlay (1:1 of 2.4% Avicel and 2× MEM, supplemented with 10% FBS) was then pipetted into each well. Cells were left to plaque for four days post-infection. The Avicel overlay was removed with two 1-mL PBS washes before cells were fixed and stained with 1 mL 0.5% crystal violet (in a 1:1 solution of methanol and water) per well. After fixing/staining for 10–15 min, crystal violet was rinsed off with water. Plaques were imaged using a ChemiDoc Touch (BioRad, Hercules, CA, USA) and counted using the FIJI distribution of ImageJ (NIH) [19].

#### 2.5.2. VSV Plaque Assay

Vero cells were seeded at a density of 2.5 × 10^5^ cells/mL in 12-well plates. The following day, media was removed and cells were inoculated with 100 μL of PDI- or mock-treated VSV (in serum-free media) for 1 h with shaking every 10 min. Inoculum was then rinsed off with 1 mL PBS and 1 mL of 1.2% Avicel overlay (1:1 of 2.4% Avicel and 2× MEM, supplemented with 10% FBS) was pipetted into each well. Cells were left to plaque for one day post-infection. Avicel overlay was removed with two 1-mL PBS washes before cells were fixed and stained with 1 mL 0.5% crystal violet (in a 1:1 solution of methanol and water) per well as described. Plaques were visualized and counted by eye.

#### 2.5.3. AdV Plaque Assay

HEK293A cells were seeded at a density of 4.5 × 10^5^ cells/mL in 12-well plates. The following day, the medium was removed and cells were inoculated with 100 μL PDI- or mock-treated AdV-GFP (in serum-free medium) for 1 h with shaking every 10 min. Inoculum was then rinsed off with 1 mL PBS, and 1 mL of pre-warmed 0.4% agarose overlay (1:4 2% agarose and complete media) was pipetted into each well. Cells were incubated for eight days post-infection to allow the development of visible plaques. GFP+ plaques were visualized and counted using a fluorescence microscope.

### 2.6. DNase-Protection Assays and qPCR

Viral DNA was extracted from 100 μL HSV-1/Orthoquin inoculum using a DNeasy Blood and Tissue Kit (Qiagen, Hilden, Germany), with some alterations to the protocol. Briefly, 80 μL of PBS were added to each HSV-1/Orthoquin tube, followed by 20 μL of DNase I (3 mg/mL; Sigma, St. Louis, MO, USA). Samples were then incubated at 37 °C for 30 min. A Buffer AL (from kit) master mix was made by combining (per sample): 200 μL Buffer AL, 5 μg salmon sperm DNA (Invitrogen, Carlsbad, CA, USA) as carrier DNA, and 100 pg luciferase vector (pGL4.26) to normalize recovery. After incubation, 20 μL Proteinase K and 200 μL Buffer AL master mix were added to each sample. Samples were mixed by vortexing and incubated at 56 °C for 10 min. The resulting mixture was then processed according to the manufacturer’s instructions.

Quantitative PCR was performed using GoTaq qPCR Master Mix (Promega, Madison, WI, USA) in 10 µL reactions, following the manufacturer’s instructions using primers to amplify a viral gene, UL54 (UL54-F 5′-TTGTCATTCTGGCCAGGCTC-3′, UL54-R 5′-TCAACTCGCAGACACGACTC-3′) and *Luc2* from the spiked-in pGL4.26 plasmid (*Luc2*-F 5′-TTCGGCAACCAGATCATCCC-3′, *Luc2*-R 5′-TGCGCAAGAATAGCTCCTCC-3′). Samples were run in duplicate in a Bio-Rad CFX connect thermal cycler with the following two-step protocol: 3 min at 95 °C, 39 cycles of 10 s at 95 °C and 30 s at 60 °C. A melt curve was completed from 65 °C to 95 °C with a read every 5 s. Product specificity was determined through single PCR melting peaks. Data were analyzed using the ΔΔCt method; gene expression was normalized to luciferase plasmid and expressed as fold change over the “dark” 10^−6^ sample.

### 2.7. Immunoblotting

HeLa cells seeded into 15-cm dishes were mock infected or infected with HSV-1 at an MOI of 3 and were left in DMEM containing 1% FBS for 48 h. Extracellular virus was obtained following a modified version of the virus purification protocol published by Loret et al. [20]. Briefly, cell culture supernatants from mock or HSV-1 infected cells were centrifuged at 500× *g* for 5 min to remove cells and large debris. The samples were treated with 50 μg/mL DNase I for 30 min on ice followed by two sequential centrifugation steps at >16,000× *g* for 30 min at 4 °C. Any pelleted material was resuspended in serum-free DMEM. Extracellular virus titer was determined via plaque assay on HeLa cells overlaid with DMEM containing 1% human serum, penicillin, streptomycin, and l-glutamine.

Approximately 5.0 × 10^5^ PFU of extracellular HSV-1 (or equivalent volume of mock sample) were diluted in serum-free DMEM and were left untreated, treated with vehicle control (DMSO), or treated with the indicated concentrations of Orthoquin and incubated in “light” or “dark,” as indicated in Section 2.3. After light exposure, Laemmli buffer was added and the samples were boiled prior to SDS-PAGE and immunoblotting. The PVDF membranes were blocked using Tris-buffered saline containing 0.1% Tween 20 (TBST) and 5% skim milk and then incubated with either rabbit anti-HSV antibody (Abcam, ab9533, Cambridge, MA, USA) and anti-rabbit HRP-linked secondary antibody (Cell Signaling, 7074, Danvers, MA, USA) or rabbit anti-β-actin-HRP conjugate antibody (Cell Signaling, 5125). All antibodies were diluted in TBST containing 1% skim milk. The blots were developed using Clarity Western ECL Blotting Substrate (BioRad) and proteins were visualized using a ChemiDoc Imaging System (BioRad).

### 2.8. Statistics

All normalization and data management was performed with Microsoft Excel for Mac (Microsoft, version 16.17, Redmond, WA, USA). Significant statistical differences between light and dark treatments were determined by two-way ANOVA with Sidak’s multiple comparison test using Prism 7 (GraphPad, La Jolla, CA, USA). *p*-values < 0.05 are depicted on the graph by an asterisk (*).

## 3. Results

### 3.1. Description and Preparation of Orthoquin

Orthoquin is a plant-derived natural product extract from the root of *Polygonum cuspidatum*, a perennial plant species that is known for its spreading rhizomes, reddish-brown stems, petioled leaves, and white flowers in drooping panicles. This particular root and its various extracts are officially listed in the Chinese Pharmacopoeia [21], and have been used in traditional Chinese medicine for treating a variety of maladies. *Polygonum cuspidatum* is thought to have originated in China and later migrated to Japan, but today it is found throughout North America and is viewed as an invasive species (often referred to as Japanese Knotweed).

**Figure 1:**
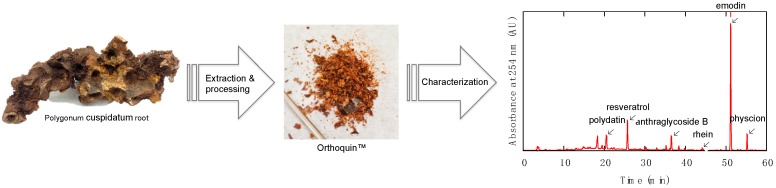
Overview of the preparation and characterization steps for producing Orthoquin^TM^.

The preparation of Orthoquin used for the present study was derived from *Polygonum cuspidatum* root harvested in Nova Scotia, Canada. Briefly, roots were washed to remove excess soil, dried, chipped, dried again, and then extracted with 100% ethanol at room temperature for several weeks. The ethanolic solution containing the extract was concentrated in vacuo to give the bioactive product, which was further enriched by a proprietary fractionation process. The final product was characterized by HPLC (Figure 1) based on UV-Vis absorption signals at five different wavelengths (254 nm, 306 nm, 320 nm, 435 nm, and 450 nm). The largest identifiable signals based on reference standards were due to polydatin (63 µg mg^−1^), resveratrol (60 µg mg^−1^), anthraglycoside B (12 µg mg^−1^), rhein (5.6 µg mg^−1^), emodin (50 µg mg^−1^), and physcion (10 µg mg^−1^).

### 3.2. Measurement of Phototoxic Threshold of Orthoquin on HeLa and hTert-BJ Cells

**Figure 2:**
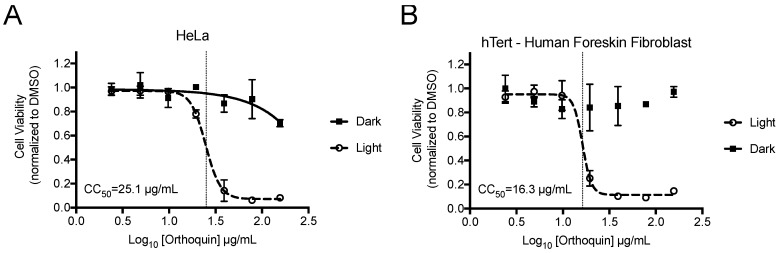
Establishment of phototoxic threshold of Orthoquin on HeLa (**A**) and hTert-immortalized human BJ cells (**B**). Cells were seeded in a 96-well plate and treated the following day with Orthoquin or DMSO vehicle control. At 16 h post-treatment, plates were exposed to a visible LED light for 30 min, with the dark plate wrapped in aluminum foil. alamarBlue^®^ cell viability assay was performed 48 h post-treatment. *n* = 3 ± SEM independent biological replicates. CC_50_ values, denoted with a vertical line, were calculated using Prism 7.

alamarBlue cell viability assays were performed to identify phototoxic threshold concentrations of Orthoquin on transformed HeLa epithelial cells and non-neoplastic immortalized hTert-BJ fibroblast cells. Cells were treated with Orthoquin or vehicle control followed by exposure to light (or a sham dark treatment) for 30 min at room temperature. The 50% phototoxic concentration (CC_50_) of Orthoquin in HeLa and hTert-BJ cells was 25.1 and 16.3 µg/mL, respectively, with no substantial dark cytotoxicity over the concentration range tested (Figure 2). Concentrations of 1 µg/mL or lower of Orthoquin were used in all subsequent infection experiments (except where noted in Figure 8), which fall well below the CC_50_ value for both cell lines.

### 3.3. Differential Baseline Photosensitivity of HSV-1 and HSV-2

**Figure 3:**
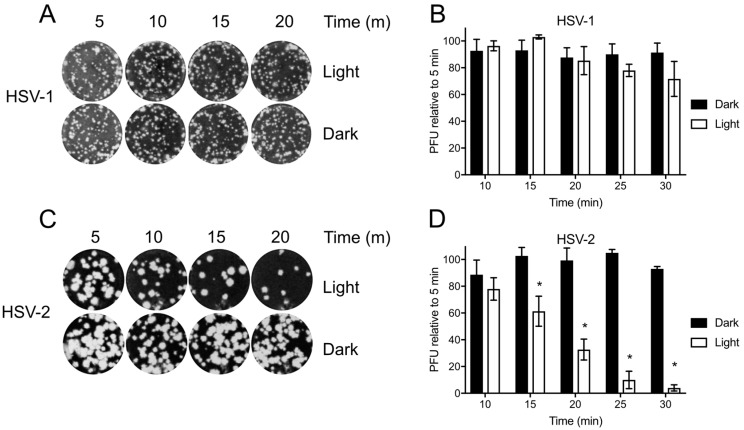
Differential sensitivity of HSV-1 and HSV-2 to white light. Viral inoculum was diluted and exposed to a 65 W LED light in clear (light) or black (dark) tubes for the indicated times, and used to infect HeLa cell monolayers as described in the methods. (**A**) Representative image of HSV-1 plaque assay, quantified in (**B**). (**C**) Representative HSV-2 plaque assay, quantified in (**D**). Values in (**B**,**D**) are normalized the number of PFUs detected after 5 min of light exposure. *n* = 3 ± SEM from three independent biological replicates. * = *p* < 0.05 by two-way ANOVA.

Before testing Orthoquin PDI, HSV-1 and HSV-2 preparations were first exposed to light to determine the baseline levels of photosensitivity. Inocula were exposed to light for 5–30 min and used to infect monolayers of HeLa cells for plaque assays. HSV-1 plaque formation was not significantly impaired due to exposure to light (Figure 3A,B); however, a marked decrease in HSV-2 plaque formation was observed after 15 min of light exposure, and plaque formation was almost completely inhibited after 30 min of light exposure (Figure 3C,D). Because light alone had little effect on HSV-1, we further investigated the effect of Orthoquin PDI on viral replication predominantly in the HSV-1 infection model.

### 3.4. Orthoquin Exhibits Light-Dependent Antiviral Activity against HSV-1 and HSV-2

**Figure 4:**
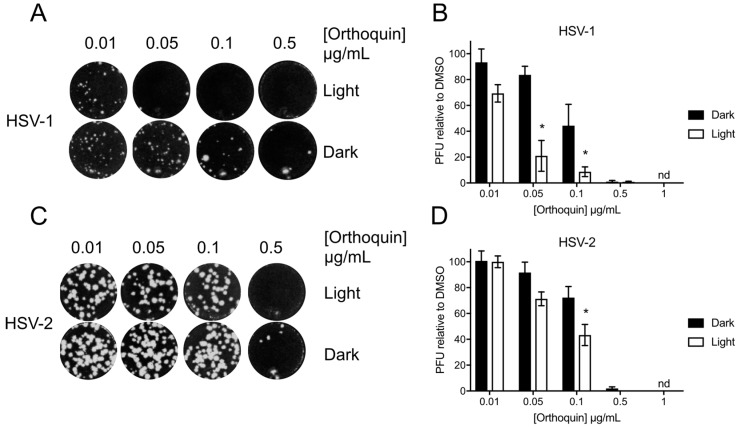
Light-dependent reduction of HSV-1 and HSV-2 plaque formation with Orthoquin treatment. HSV-1 and HSV-2 inocula were mixed with Orthoquin at concentrations ranging from 0.01 to 1 μg/mL and exposed to a 65 W LED light in clear (light) or black (dark) tubes for 10 min (HSV-1) or 5 min (HSV-2). Treated inocula were used to infect HeLa cell monolayers, as described in the methods. (**A**) Representative image of HSV-1 plaque assay, quantified in (**B**). (**C**) Representative HSV-2 plaque assay, quantified in (**D**). Values in (**B**,**D**) are normalized to the number of PFUs detected in DMSO, light- or dark-treated wells. *n* = 3 ± SEM from three independent biological replicates. * = *p* < 0.05 by two-way ANOVA. nd = none detected.

The antiviral activity of Orthoquin was assessed at sub-cytotoxic concentrations from 0.01 to 1 μg/mL. HSV-1 and HSV-2 incolula were mixed with the indicated concentrations of Orthoquin and exposed to light for 10 min and 5 min, respectively. A significant light-dependent reduction in plaque formation was observed at 0.05 and 0.1 μg/mL doses of Orthoquin for HSV-1 and at the 0.1 μg/mL dose of Orthoquin for HSV-2 (Figure 4). However, doses of 0.5 and 1 μg/mL of Orthoquin potently inhibited HSV-1 and HSV-2 infection in a light-independent manner. Together, these findings indicate that Orthoquin posesses light-dependent anti-HSV properties, as well as light-independent properties that operate at higher concentrations. It is likely that the longer light exposure time used for HSV-1 is enhancing Orthoquin’s antiviral effects at the 0.05 µg/mL dose (relative to the same concentration for HSV-2), but we did not test HSV-2 with Orthoquin for longer than 5 min due to inherent light sensitivity.

### 3.5. Orthoquin PDI Inactivates HSV-1 across a 100-Fold Range of Inoculum Titer

**Figure 5:**
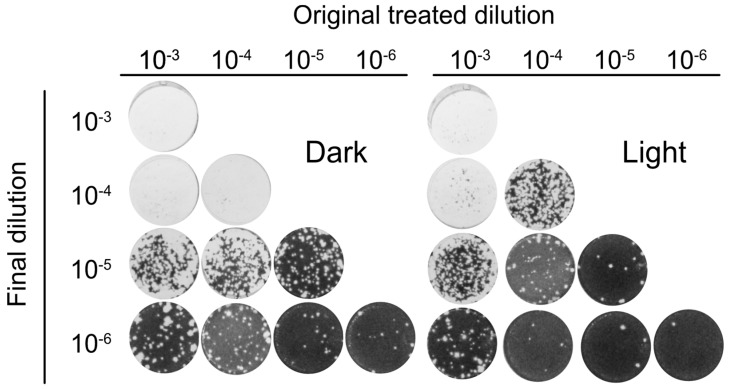
Orthoquin PDI inactivates HSV-1 across a 100-fold range of inoculum titer. Four dilutions of HSV-1 inocula (10^−3^, 10^−4^, 10^−5^, and 10^−6^) were mixed with 0.1 µg/mL Orthoquin and exposed to a 65 W LED light in clear (light) or black (dark) tubes for 10 min. Following treatment, each inoculum was diluted in multiple 10-fold dilutions to a final dilution of 10^−6^ and used to infect HeLa cell monolayers. Cells were fixed and stained as described in the methods. *n* = 3 independent biological replicates. Representative plaque assays are shown.

To further analyze the antiviral activity of Orthoquin, four 10-fold dilutions of HSV-1 virion stocks (ranging from 10^−3^ to 10^−6^) were mixed with 0.1 μg/mL Orthoquin. The samples were either treated with light or kept in the dark, were not exposed or were exposed to light, and subsequently diluted to 10^−6^ prior to inoculation of HeLa cell monolayers for plaque assays. Light-activated Orthoquin treatment reduced plaques across three 10-fold HSV-1 dilutions (10^−4^–10^−6^) but was ineffective against the highest titer inoculum (10^−3^) (Figure 5). These findings indicate that while Orthoquin PDI inactivates HSV-1 across a 100-fold range of inoculum titer, it fails to inactivate highly-concentrated inoculum.

### 3.6. Orthoquin PDI Depends on Direct Contact with HSV-1 Virions

**Figure 6:**
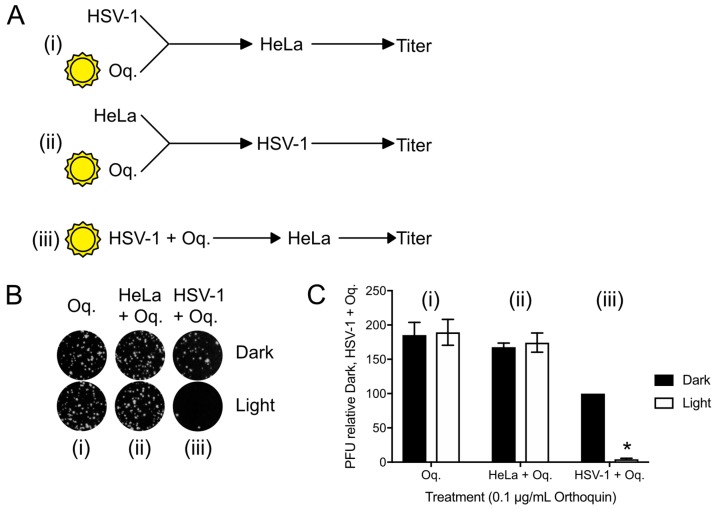
Orthoquin anti-HSV-1 activity depends on close proximity with virions during the photoactivation step. (**A**) Experimental schema for Orthoquin treatments: (i) Orthoquin was exposed to light in clear or dark tubes for 10 min and was then combined with HSV-1 virions and used to infect HeLa cells; (ii) Orthoquin was exposed to light in clear or dark tubes then used to treat a monolayer of HeLa cells for 1 h. Afterwards, the cells were infected with HSV-1; (iii) Orthoquin PDI of virions prior to infection as in Figure 3 and Figure 4. (**B**) Representative plaque assays are shown for the three experimental schema. (**C**) Plaque assay data from three independent experiments were quantified. Values were normalized to the number of plaques detected to the combined Orthoquin/HSV-1 treatment in the dark tubes (schema iii). *n* = 3 ± SEM from three independent biological replicates. * = *p* < 0.05 by two-way ANOVA. Oq = Orthoquin.

To confirm that the antiviral effect of Orthoquin was due to PDI effects on virions, plaque assays with three different Orthoquin treatments were performed; (i) Orthoquin was exposed to light before combining with HSV-1 incolula, and then infecting a HeLa monolayer; this treatment would allow us to determine whether Orthoquin antiviral activity was due to a photoproduct not acting through PDI; (ii) Orthoquin was exposed to light before treating a HeLa cell monolayer with it for 1 h, rinsing it off and inoculating the cell monolayer with HSV-1; this treatment would allow us to test whether a photoproduct of Orthoquin primes a light-independent antiviral response in cells before inoculation with HSV-1; or (iii) Orthoquin was mixed with HSV-1 and then exposed to light, followed by infection of a HeLa monolayer (as in Figure 4 and Figure 5). As expected for Orthoquin-mediated antiviral PDI, treatments (i) and (ii) did not reduce plaque formation whereas (iii) was effective at reducing plaque formation in a light-dependent manner (Figure 6). These findings confirm that Orthoquin exerts its antiviral activity through PDI, which requires the close proximity of Orthoquin with target virions during light exposure. 

### 3.7. Orthoquin PDI Does Not Release HSV-1 Genomic DNA from Virions

**Figure 7:**
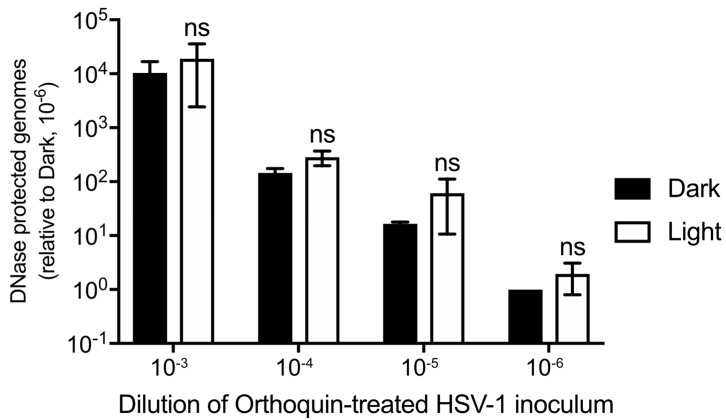
Orthoquin does not damage HSV-1 capsids. Four 10-fold dilutions of HSV-1 inocula were mixed with 0.1 μg/mL Orthoquin and exposed to a 65 W LED light in clear (light) or black (dark) tubes for 10 min. Following treatment, samples were treated with DNase I for 30 min at 37 °C. Viral DNA was then extracted in lysis buffer and qPCR was performed using primers amplifying UL54 (viral DNA) and luciferase (recovery control). The data shown are means ± standard deviation of three independent experiments. *n* = 3 ± SD from three independent biological replicates; ns = non-significant by two-way ANOVA.

Orthoquin PDI is known to produce ROS, which could physically disrupt structural components of HSV-1 virions, including envelope glycoproteins and lipids, capsid proteins and nucleic acids. To determine whether Orthoquin PDI inhibits HSV-1 by causing capsid damage, four 10-fold dilutions of HSV-1 virion stocks were treated with 0.1 μg/mL Orthoquin, exposed to light and then incubated with DNase I. DNA was then extracted and a viral gene, UL54, was amplified by qPCR. If the capsid was compromised by Orthoquin treatment, no amplification of viral DNA would be expected because DNase I penetratration of the capsid would degrade viral DNA. A ~10-fold difference in viral gene amplification was observed between each 10-fold HSV-1 dilution, indicating that the assay reliably amplified viral DNA (Figure 7). Within dilutions, no significant difference in viral gene amplification was observed between “light” and “dark” treatments, indicating that Orthoquin treatment does not compromise HSV-1 capsid integrity.

### 3.8. High Doses of Orthoquin Inhibit Detection of HSV-1 Structural Proteins

**Figure 8:**
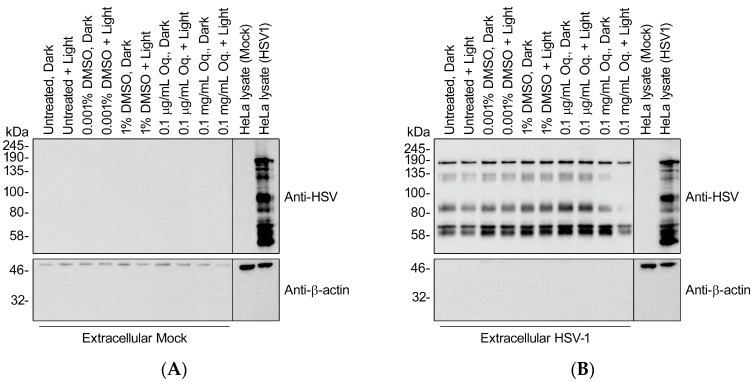
High doses of Orthoquin inhibit detection of HSV-1 structural proteins. Culture supernatants from mock-infected HeLa cells (**A**) or HSV-1-infected HeLa cells (**B**) were processed by differential centrifugation and exposed to 0.1 μg/mL or 0.1 mg/mL Orthoquin, or DMSO vehicle control, or mock-treated for 10 min and exposed to a 65 W LED light in clear (light) or black (dark) tubes. Following treatment, HSV-1 particle preparations were subjected to SDS-PAGE and immunoblotting with a rabbit polyclonal pan-anti-HSV1 antibody that detects viral structural proteins. Uninfected and infected HeLa cell lysates were included as controls for HSV-1 antigen detection.

Previous reports of PDI of herpesviruses suggested defects in cell adsorption and entry, which requires intact envelope glycoproteins [22]. To determine whether Orthoquin PDI disrupts HSV-1 glycoproteins, we isolated extracellular viral particles by differential centrifugation and treated them with 0.1 μg/mL (effective dose used in Figure 3, Figure 4, Figure 5 and Figure 6) or 0.1 mg/mL Orthoquin in vitro for 10 min in the presence of light, prior to processing samples for SDS-PAGE and immunoblotting with a pan-anti-HSV-1 antibody that detects HSV-1 glycoproteins (Abcam, Inc., technical support). Immunoblotting crude HSV-1-infected HeLa cell lysates confirmed detection of diverse HSV-1 antigens, some of which were also clearly evident in the viral particle samples, indicating that they represent structural proteins (Figure 8). The pattern of detectable protein species from viral particle samples was unchanged by treatment with 0.1 μg/mL Orthoquin. By contrast, treatment with the higher 0.1 mg/mL dose of Orthoquin caused a dramatic loss of detection of certain antigens in the 80–135 kDa range, in a light-dependent manner. We do not know whether this loss of antigen detection is due to Orthoquin PDI-mediated degradation of select HSV-1 proteins, or physical disruption of discrete antigenic sites, or other mechanisms, and this 0.1 mg/mL dose far exceeds the effective dose previously established in Figure 3. Nevertheless, these findings suggest that Orthoquin PDI can physically disrupt HSV-1 structural proteins. Modest physical disruption of HSV-1 structural proteins (i.e., envelope glycoproteins) by treatment with 0.1 μg/mL Orthoquin may be sufficient to inhibit infectivity without causing gross changes in structural proteins that would be detectable in an immunoblotting experiment.

### 3.9. Orthoquin PDI of Vesicular Stomatitis Virus and Adenovirus

**Figure 9:**
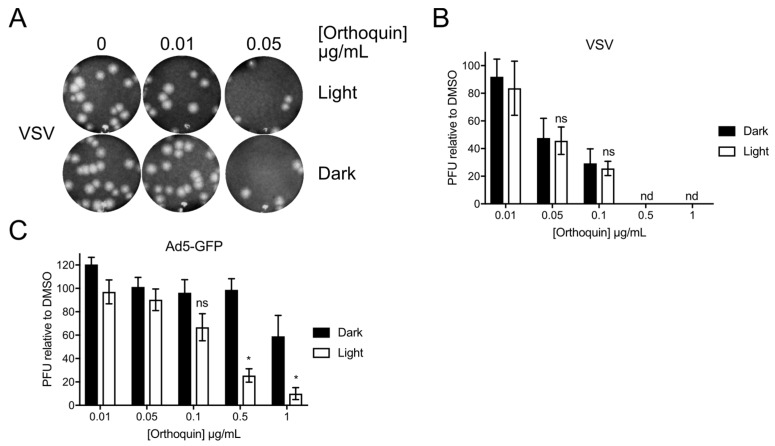
Orthoquin inhibits vesicular stomatitis virus (VSV) and human adenovirus type 5 (AdV) plaque formation. (**A**) VSV inocula were mixed with Orthoquin at concentrations ranging from 0.01 to 1 μg/mL and exposed to a 65 W LED light in clear (light) or black (dark) tubes for 5 min. Treated inocula were then plaqued on a monolayer of Vero cells, as described in the methods. A representative image of VSV plaques is shown. (**B**) Quantification of plaques in (**A**). Values were normalized to the number of PFUs detected in DMSO, light or dark treated wells. *n* = 3 ± SEM from three independent biological replicates. (**C**) AdV inocula were mixed with Orthoquin at concentrations ranging from 0.01 to 1 μg/mL and exposed to a 65 W LED light in clear (light) or black (dark) tubes for 10 min. Treated-inocula were then plaqued on monolayers of HEK293A cells, as described in the methods. Plaques were counted using a fluorescent microscope. *n* = 4 ± SEM from four independent biological replicates. * = *p* < 0.05 by two-way ANOVA. ns = non-significant by two-way ANOVA. nd = none detected.

To determine whether Orthoquin can inactivate other viruses, we attempted PDI of vesicular stomatitis virus (VSV), an enveloped virus of the family Rhabdoviridae, and human adenovirus (AdV), a non-enveloped virus of the family Adenoviridae. VSV and AdV inocula were incubated with 0.01 to 1 μg/mL Orthoquin in clear or opaque (black) tubes, followed by infection of target host cells. A sharp decrease in VSV plaques was observed starting at 0.05 μg/mL Orthoquin, but light exposure did not improve antiviral activity at any concentration (Figure 9A,B). By contrast, a significant light-dependent reduction in AdV plaque formation was observed starting at 0.5 μg/mL Orthoquin (Figure 9C). Thus, while Orthoquin displayed antiviral activity against these viruses, the AdV was relatively resistant to Orthoquin compared to HSV-1 and HSV-2, and inactivation of VSV may involve a light-independent mechanism of action.

## 4. Discussion

Orthoquin is a botanical extract with antibacterial properties that are amplified by light exposure [17]. Consistent with past PDI studies with different photosensitizing agents [23], we show that Orthoquin inhibits HSV-1 and HSV-2 infection in a light-dependent manner. Surprisingly, photosensitivity tests revealed a high baseline photosensitivity of HSV-2, with almost complete inhibition of plaque formation after 30 min of light exposure. By contrast, HSV-1 was relatively photoresistant. For this reason, we focused mechanistic studies on HSV-1. We showed that anti-HSV-1 PDI required co-administration of Orthoquin and light to viral inoculum, whereas pre-exposure of target cell monolayers to Orthoquin and light had no effect at the doses used in this study. This is consistent with known properties of photosensitizing agents that generate short-lived ROS and singlet oxygen that can damage nearby macromolecules like proteins, lipids and nucleic acids [24] and indicates that PDI requires close proximity to its target for its antiviral effect.

Improved understanding of the properties of Orthoquin will inform future investigations of the mechanism of virion damage. Orthoquin contains two compounds with previously reported antiviral activity, resveratrol and emodin. Resveratrol inhibits multiple enveloped and non-enveloped viruses by modulating signal transduction pathways (reviewed in [25,26]) and emodin was observed to inhibit HSV-1 [27], human cytomegalovirus [28], hepatitis B virus [29], influenza A virus (IAV) [30], and human immunodeficiency virus type 1 replication [31]. Emodin has also been shown to suppress efficient reactivation of Epstein‒Barr virus from latency [32], poliovirus-induced cytopathic effect [33], severe acute respiratory syndrome coronavirus spike protein attachment [34] and HSV-1 alkaline exonuclease activity [35]. One known function of emodin is to act as a competitive inhibitor of casein kinase II [36], a cellular kinase that plays key supportive roles in many viral infections [37,38,39,40]. Additionally, as a highly hydrophobic anthraquinone, emodin invades phospholipid bilayers and promotes non-lamellar phase transitions [41]. Our data demonstrate that Orthoquin displays maximal inhibitory activity when in close proximity with enveloped virions prior to infection, which raises the possibility of emodin-induced phase changes in the viral envelope concomitant with PDI.

We were unable to pinpoint the molecular target(s) of Orthoquin PDI on HSV-1 virions in this study, but high doses of photoactivated Orthoquin prevented a pan-anti-HSV-1 polyclonal antibody from detecting certain structural protein components of purified HSV-1 virions. This antibody detects major HSV-1 glycoprotein antigens, suggesting that Orthoquin PDI-generated ROS may physically disrupt antigens to prevent detection, alter protein mobility in SDS-PAGE, and/or accelerate protein degradation. Indeed, another anthraquinone derivative related to emodin, hypericin, was shown to induce protein cross-linking in vitro through singlet oxygen production following photactivation [42]. While our data does not demonstrate the presence of cross-linked viral proteins in Orthoquin-treated HSV-1 virions, it would nevertheless be of interest to investigate any changes to virion proteins at the molecular level. Our study also suggests that Orthoquin PDI leaves the underlying HSV-1 nucleocapsids intact, as viral dsDNA genome content was unchanged in treated inoculum compared to untreated controls. In an attempt to identify specific glycoproteins disrupted by Orthoquin PDI, we performed additional immunoblotting experiments with antibodies raised against HSV-1 glycoprotein C; however, these experiments were inconclusive because these antibodies were not sensitive enough to detect glycoprotein C in our purified virion preparations.

Because the lipid bilayer that comprises the HSV envelope is a potential target of PDI, we tested Orthoquin against an additional enveloped virus (VSV) and a non-enveloped virus (AdV). VSV was susceptible to Orthoquin in a light-independent manner, suggesting a potentially distinct mechanism of action. By contrast, AdV was susceptible to Orthoquin in a light- and dose-dependent manner, and only slightly less sensitive than HSV-1 and HSV-2. Previous research showed that emodin inactivated enveloped viruses (herpesviruses, Japanese encephalitis virus, and IAV) but had no effect on non-enveloped viruses AdV serotype 37 and rhinovirus serotype 1A [43,44]. However, in the study by Lin, et al. emodin inhibited enterovirus replication by stimulating interferon production [44]. Similarly, resveratrol has been shown to inhibit IAV replication by stimulating interferon production [30]. Because AdV lacks a lipid envelope and was maximally inhibited in the presence of light, we speculate that Orthoquin interferes with the function of surface-exposed viral proteins; however, we cannot exclude a potential role for interferon in Orthoquin antiviral activity at this time.

Acyclovir is the most commonly used HSV antiviral. Though resistance to acyclovir is clinically insignificant in otherwise healthy individuals (0.1–0.7%) [5], immunosuppressed individuals have much higher incidences (4–10%). The prevalence of resistance is very strongly linked to the degree of immunosuppression and duration of acyclovir exposure [45]. These issues highlight the important role that host-targeted or broadly-acting antivirals could play in treatment of drug-resistant HSV infections.

More research will be required to determine the precise anti-HSV mechanism of action of Orthoquin. Because Orthoquin contains multiple known antiviral molecules, it may directly inactivate virions in the inoculum via PDI, while disrupting downstream intracellular steps of viral infection as well. We speculate that Orthoquin may provide a viable broadly-acting alternative for inactivation of HSV in surface lesions, which may be effective against current drug-resistant strains and mitigate the emergence of resistance.
